# Corporate social responsibility of pharmaceutical industry in Korea

**DOI:** 10.3389/fphar.2022.950669

**Published:** 2022-08-23

**Authors:** Green Bae, Jeong-Hoon Ahn, Kyung-Min Lim, SeungJin Bae

**Affiliations:** College of Pharmacy, Ewha Womans University, Seoul, South Korea

**Keywords:** CSR, social contribution, philanthropic activities, pharmaceutical company, ESG

## Abstract

**Background:** Global pharmaceutical companies in Korea argue that the development of innovative drugs should be recognized as a social contribution, yet it has been countered by various stakeholders. The need to distinguish between philanthropic activities and Corporate Social Responsibility (CSR) of pharmaceutical companies and reaching consensus in the Korean context has been raised. We sought to evaluate the CSR status of Korean pharmaceutical companies and collect the stakeholders’ opinions to define philanthropic activities and CSR related to pharmaceutical companies in Korea.

**Methods:** We conducted a literature review on the definition of CSR of pharmaceutical companies, and the CSR activities of the domestic pharmaceutical companies were compared with those of global pharmaceutical companies operating in Korea. The opinions of stakeholder groups (patient advocate groups, consumer organizations, and domestic/global pharmaceutical companies) were collected using focus group interviews (FGI) and written surveys.

**Results:** Literature review suggested that CSR is categorized as “must do” (economic and legal responsibilities), “ought to do” (ethical responsibilities), and “can do” (philanthropic responsibilities), whereas contributions beyond the economic, legal, or ethical responsibilities can be defined as “can do” (philanthropic responsibilities). Domestic pharmaceutical companies simply adopted systems for ethical and ESG (Environmental, Social, and Governance) management, which are at the “ought to do” level (ethical responsibility), whereas the headquarters of these global pharmaceutical companies established the CSR team and systematically reported on the CSR activity, including ESG management reports, which is at the “ought to do” level and further moving to the “can do” level, but the Korean branch rarely has CSR teams, and the CSR activities in Korea were also insufficient. At the FGI, the global pharmaceutical companies argued that CSR activities, such as innovative drug development, should be recognized as similar to philanthropic activities, yet stakeholders besides them suggested that those activities are “can do” rather than being philanthropic.

**Discussion:** We found that the pharmaceutical companies in Korea are attempting to achieve the “ought to do” level (ethical responsibilities) while complying with the “must do” level (legal and economic responsibilities) yet not philanthropic activities. A social consensus regarding the philanthropic responsibilities of pharmaceutical companies in Korea was not reached.

## 1 Introduction

The European Commission defined CSR as “a concept whereby companies integrate social and environmental concerns in their business operations and in their interaction with their stakeholders on a voluntary basis” (Ankersmit, 2020). Through CSR, companies can create conditions favorable for sustainable growth and employment generation in the medium and long term (Chhaparia and Jha, 2018).

In 2017, the definition of pharmaceutical companies’ social contribution was heavily discussed in Korea since global pharmaceutical companies in Korea argued that the development of innovative new drugs is the most important social contribution of the pharmaceutical industry, and thus, innovative new drugs should get a higher price than the conventional pricing method. However, it is unclear whether developing innovative drugs is a pharmaceutical company’s ethical responsibility, which it is required to do, or a philanthropic activity, as insisted by the pharmaceutical industry. Moreover, the definition of the corporate social responsibility (CSR) of pharmaceutical companies in the Korean context has not been fully discussed.

CSR has been recognized as a part of a company’s business strategy. Although CSR activity has already been established and actively performed by global pharmaceutical companies, it has not been actively conducted by domestic companies. The EU ordered for the mandatory disclosure of CSR information for companies with 500 or more employees, (Veltri et al., 2020), and approximately 3,040 companies in China publish sustainability reports (Wong, 2020). However, among Korean pharmaceutical companies, only one company published its CSR report for the first time in 2018 (Hanmi, 2018). Until now, the production of generics has been the main source of revenue for Korean pharmaceutical companies, and because of the small market, which accounts for only 2% of the global pharmaceutical and biomarket, they have been rather passive in R&D and CSR (Kim et al., 2017).

Along with maximizing profits, the core idea of CSR is for companies to pursue other prosocial goals. This article focuses on the social contribution of the CSR subsection, and it does not address the other subsections, economy, and environment. Based on the CSR classification, we analyzed and compared the CSR activities of the domestic and global pharmaceutical companies in Korea. Also, we surveyed the opinions of stakeholders about CSR and conducted focus group interviews (FGI) with domestic/global pharmaceutical companies, consumer organizations, patient groups, and academic experts. Through this study, we attempted defining CSR related to pharmaceutical companies in Korea and distinguish between philanthropic contributions and the CSR of pharmaceutical companies in the Korean context.

## 2 Materials and methods

This study used a mixed method. We collect data for analyzing the CSR activities of pharmaceutical companies in Korea and analyze their activities by applying the analysis framework derived through the literature review as a case study. In addition, we conducted a FGI with stakeholders to investigate the categories of CSR activities that could be considered in the context of drug prices.

### 2.1 Analytical framework of corporate social responsibility in the pharmaceutical industry

Global pharmaceutical companies in Korea argue that a unique social contribution rendered by the pharmaceutical industry is the development of innovative new drugs in the field where treatments are not available. However, Joseph E. Stiglitz and Arjun Jayadev highlight that the current pharmaceutical drug discovery model is quite inefficient as the drug price for the public is high, R&D through public funds is privatized, and patents act as a barrier to knowledge (Stiglitz and Jayadev, 2010). The reasons include excessive generic production, several intellectual property rights (patent) acting as a knowledge barrier, the privatization of publicly funded R&D outcome, and the lack of R&D of disease with weak purchasing power. Also, criticism exists that pharmaceutical companies have been heavily rewarded for their marginal innovations, such as incrementally modified drugs (super generic) and generics, since the demand for pharmaceuticals is inelastic (Ganuza et al., 2009). Starting with Nordhaus (1969), many researchers argue that underinvestment in R&D is likely to occur unless the surplus profits generated by companies are necessarily invested in development, and they refuted the claim that operating profit is an incentive for innovation. Until now, researchers argued that there may have been incorrect resource allocation in terms of investments in small-scale innovations, such as incrementally modified drugs (super generic) or generics, rather than investments in innovative new drug development.

The government, the media, and NGOs are putting pressure on the CSR of pharmaceutical companies (King et al., 2017). CSR has become more important because of public criticism and distrust of pharmaceutical companies on the issue of “lack access to essential medicines in the third world, high pricing, high level of profit, animal testing, and other related problems.” Pharmaceutical companies should be more sensitive to CSR than other industries, and to gain social recognition, pharmaceutical companies must effectively cooperate to respond to social, economic, and environmental obligations. Corporate social responsibility may vary depending on the CSR definition, but common CSR activities of global pharmaceutical companies are strengthening drug distribution infrastructure, giving discounts to developing countries, and conducting R&D for underdeveloped disease areas (Droppert and Bennett, 2015). According to a paper by (Brammer and Pavelin, 2005), pharmaceutical companies ranked first to fourth in the amount of donations from the top 25 community-contributing companies surveyed in the United States Brammer and Pavelin, (2005). Also, the UK’s community contribution placed pharmaceutical companies at the top. Because of analyzing the amount of local community contribution by the business sector and the proportion of sales, the pharmaceutical sector was ranked at the top in the United States and the United Kingdom for philanthropic activities among the CRS classification.

**FIGURE 1:**
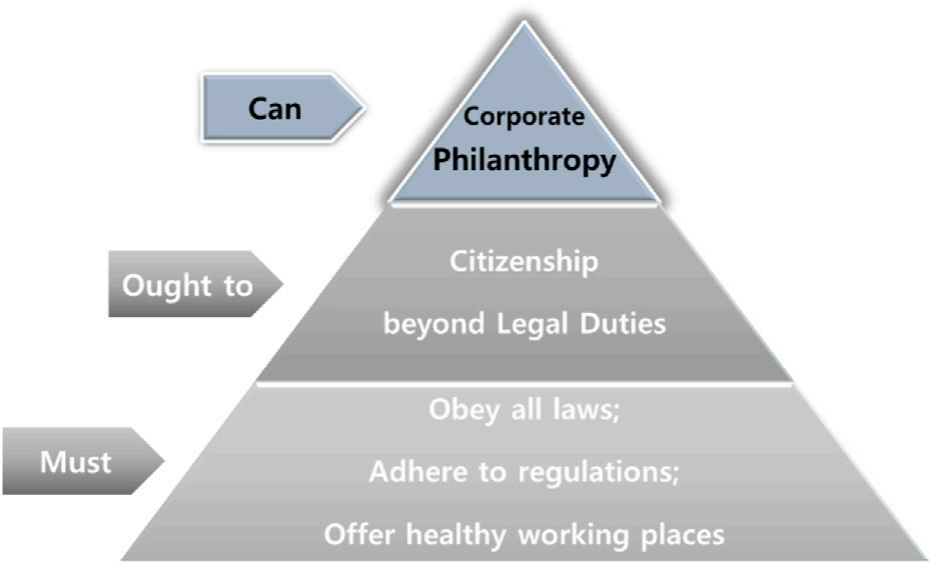
The classification of Corporate Social Responsibility. Source: Reorganize “the hierarchy of corporate responsibilities” in Leisinger (2005). The corporate social responsibility of the pharmaceutical industry: idealism without illusion and realism without resignation. Business Ethics Quarterly, 577-594.

CSR, or corporate citizenship, is based on the idea that a company is socially responsible to the public and is cautious that its business ethics can demonstrate a positive economic impact on the companies (Joyner and Payne, 2002). Leisinger says that the company complies with the law to provide a healthy environment for its employees and local people, minimize pollution, and contribute to the national economy, with the profits generated by successful R&D classified as a “must do” level for the CSR (Figure 1). It was classified as “ought to do” that requires compliance with the global principle, such as The Global Compact Principle presented by the UN, beyond the scope of raw materials required by companies. The Global Compact Principle proposed by the UN comprises Human Right, Labor Standards, Environment, and Anti-Corruption. Next, philanthropy activities that are customarily required as desirable behaviors are classified as “can do” (Figure 1).

According to Carroll (1999), the responsibilities and characteristics that the society demands from corporations are largely defined as economic and legal responsibilities (must do), ethical responsibilities (ought to do), and philanthropic responsibilities (can do) (Carroll and Buchholtz, 2014).

The legal and economic responsibility of pharmaceutical companies is considered as “must do,” since it specifies meeting legal standards (obey the regulation), providing economically sound products, effectively meeting buyers’ demands, providing products and services at competitive prices, and contributing for the public’s benefit (Carroll and Buchholtz, 2014). It not only considers profitability but also aims to improve the quality of life of patients, which is a “must do” area. In particular, the production of orphan drugs with affordable price can be regarded as an economic responsibility, that is, “must do” (Carroll and Buchholtz, 2014). In addition, it includes complying with the obligation not to act contrary to responsibility for compliance with the distribution system and sales order (Leisinger, 2005).

The ethical responsibilities of pharmaceutical companies are considered as “ought to do” and include making donations in cases of acute emergency disease (infectious diseases, etc.) or demonstrating a flexible attitude in negotiating the supply of essential medicine, for example, setting lower prices for poor patients in developing countries (Carroll and Buchholtz, 2014). However, in this case, preventing the re-export or leakage of low-cost medicines is necessary, and an appropriate political environment and agreement with developed countries are required. Pharmaceutical companies demonstrate a responsibility and social expectations to provide drugs at an affordable price. Therefore, even if the market exhibits a monopolistic character, absurdly expensive pricing policies can be criticized. However, even orphan drugs may demonstrate a high-risk but monopolistic structure; thus, a reasonable price that does not impede accessibility is a corporate ethical responsibility and is at the “ought to do” level Leisinger, (2005).

The philanthropy activities of pharmaceutical companies have a meaning of “without any expectation of economic benefit” among the Archie Carroll’s category falls into “Can do.” They can be described as active, voluntary, and nonreciprocal efforts (organizational, financial, human resources, etc.) Leisinger, (2005). This means that necessary-oriented activities benefit humans or meet unmet social needs regardless of the specific “return on investment” to donors Leisinger, (2005).

Therefore, we would like to analyze the CSR activities of pharmaceutical companies by applying the abovementioned three classifications: economic and legal responsibility (what to do), ethical responsibility (to do), and philanthropic responsibility (what can be done).

Source: Reorganize “the hierarchy of corporate responsibilities” in (Leisinger, 2005). The corporate social responsibility of the pharmaceutical industry: idealism without illusion and realism without resignation. Business Ethics Quarterly, 577–594.

### 2.2 Case study (Corporate social responsibility activities of pharmaceutical industry in Korea)

This study compared and analyzed the CSR activities of the top seven global pharmaceutical companies in terms of sales in Korea in 2019 along with the domestic pharmaceutical companies with sales exceeding KRW 1 trillion (about USD 0.9 Billion). This study was based on 2019 data to remove the factors affecting the special situation of COVID-19. The Korean branches of Novartis, AstraZeneca, Sanofi, Roche, Pfizer, Bayer, and GlaxoSmithKline, with annual sales between KRW 300 billion and 400 billion (USD 265–353 million), were included Kim, (2020a). As domestic companies with sales of over KRW 1 trillion, Yuhan Corporation, GC Pharma, Guangdong, Celltrion, Hanmi Pharmaceutical, Daewoong Pharmaceutical, and ChongKunDang were included Kim (2019b).

To compare the CSR activities of global pharmaceutical companies and domestic pharmaceutical companies, various documents, homepages of pharmaceutical companies, public announcement documents, and the CSR report of Korean Research-based Pharmaceutical Industry Association (KRPIA) and Korea Pharmaceutical and Bio-Pharma Manufacturers Association (KPBMA) were referred to. Through literature analysis, we analyzed whether a CSR category and explanations on the website related to CSR exist, whether fair trade self-compliance (CP) has been implemented, whether a dedicated CSR team is organized, and whether a CSR report based on Global Reporting Initiative (GRI) is issued along with ESG (Environmental, Social, and Governance) grades.

### 2.3 Focus group interview

A FGI was conducted twice, which was composed of stakeholders, such as pharmaceutical companies, academia, and a consumer organization and patient group, to hear and collect various opinions on the CSR of pharmaceutical companies. Doctors who are prescribers in Korea were excluded because cases where they were suspected to be beneficiaries of pharmaceutical companies’ CSR activities in rebates were found. On 18 April 2017, 9 individuals related to the pharmaceutical industry participated, including 5 members of the KRPIA, which is mainly composed of global companies, and 4 members of the KPBMA, mainly domestic. On 19 April 2017, the second FGI comprising consumer organization(s), patient groups, and academic experts was held. A total of 5 people attended. This FGI was not subject to IRB review because it does not identify personal information of participants and it collected stakeholder’s opinion in the context of the stakeholder’s association and not individual. Also, under the regulations of the Korean bioethics law, this study, which is an opinion survey related to government policy, was excluded from the IRB’s review.

In both groups, FGI progressed for approximately 2 h. The theme of discussion was the social contribution of pharmaceutical companies. Since these FGI were classified as a survey of opinions related to government policy in accordance with the Bioethics and Safety Act, it was exempt from IRB review. Participation was completely voluntary. The verbal consent was obtained from participants when making the audio recordings. Participants were not identified, and a participant number was allocated to each, which were also used for data reporting. On the transcript, each word of the participants was recorded. The CSR analysis framework applied in this study was provided to the participants, and we asked them to discuss what CSR activities correspond according to this standard.

Participants discussed the overall CSR activities as a social contribution required from pharmaceutical companies in the Korean context, and participants, including pharmaceutical companies, discussed CSR items that can be classified as the “can do” level. Based on this, we tried to collect opinions on the areas of “ought to do” and “can do,” which are expected from domestic pharmaceutical companies. Based on these discussions, we tried to identify the “ought to do” and “can do” of CSR, which are expected from pharmaceutical companies in Korea. In the literature, charity activities and free medicine support are referred to as “can do” (Leisinger, 2005), but whether it is applicable to the Korean context as well is unclear. In addition, among the “can do” items, we also discussed whether any activities that could give preferential drug prices were found.

## 3 Results

### 3.1 Current status of corporate social responsibility of Korea Pharmaceutical industry

According to the results of the 2019 Global Pharmaceutical Company’s Social Contribution Survey, the total amount of social contribution activities was 30.2 billion Korean won ($26.7 million), which was 0.58% of sales, an increase from 0.55% of the previous year Global pharmaceutical, (2020). In addition, in 2017, the global pharmaceutical companies donated more than 0.3% of their pre–tax profits to charitable causes. This is about three times higher than the average of 10 major industries in Korea (0.11%) Korean companies, (2018). However, according to latest Corporate Giving by the FTSE 100 research of the Charities Aid Foundation, global pharmaceutical companies donated 7.5% of their pre–tax profits over the last 7 years Caf. Corporate Philanthropy: Spotlight On Pharmaceuticals, (2020).

The status of Korean pharmaceutical companies’ contributions to the domestic society can be found only in the “CSR Report” for sustainable management published by Hanmi Pharm for the third year. In 2018, Hanmi Pharmaceutical published the first CSR report in the Korean pharmaceutical industry, followed by a total of three reports from 2019 to 2020. This is the first and only Korean pharmaceutical company to publish a report for sustainability management Hanmi, (2020). Next, the cumulative donation of Korean pharmaceutical companies in the third quarter of 2019 in Korea was $14 million Kim, (2019a).

In 2019, with the goal of improving the CSR awareness and expanding the base of domestic pharmaceutical companies, the Pharmaceutical Bio CSR Research Group launched, which included 15 major domestic pharmaceutical companies, four of which were the dedicated CSR team within each enterprise Kim, (2020b). Both of the Korean pharmaceutical companies and the Korean branches of global pharmaceutical companies were criticized for showing rather superficial CSR characteristics centered on their employee volunteering. We found that managing a company with the CSR concept itself has not yet taken root in the overall management of domestic pharmaceutical companies.

#### 3.1.1 Comparison of corporate social responsibility in domestic and global pharmaceutical companies in Korea

Table 1 presents the results of a comparison of the indicators that can check the CSR activities of seven Korean pharmaceutical companies and seven global pharmaceutical companies. These categories belong to “must do” or “ought to do,” as shown in Figure 1.

**TABLE 1:** Corporate Social Responsibility in domestic and global pharmaceutical companies in Korea in “ought to do.”

Firm	Capital origin	CSR section on website	Code of ethics (CP or ISO 37001)	CSR team in the firm	CSR project results	Sustainability report or GRI	CSR mission, values, principles	ESG* level Wang, (2021)
A	Korea	O	CP(AAA)/ISO 37001	O	O	O	O	A
B	Korea	O	CP/ISO 37001	O	O	-	O	B+
C	Korea	O	CP/ISO 37001	-	-	-	O	B+
D	Korea	O	CP(AA)/ISO 37001	-	O	-	O	B+
E	Korea	X	Code of ethics	-	-	-	-	B+
F	Korea	O	CP/ISO 37001	-	-	-	O	B
G	Korea	O	CP(AA)/ISO 37001	-	O	-	O	B
Novartis	Switzerland	O	O	O (G)	O (G)	O (G)	O	18.7(L)
Astrazeneca	UK	O	O (G)	O (G)	O	O (G)	O	26.7(M)
Sanofi	France	O	O (G)	O (G)	O	O (G)	O	24.4(M)
Roche	Switzerland	O	O	O (G)	O	O (G)	O	24.3(M)
Pfizer	US	O	O	O (G)	O	O (G)	O	25.3(M)
Bayer	Germany	O	O	O (G)	O	O (G)	O	32.8(H)
GSK	UK	O	O	O (G)	O	O (G)	O	21.6(M)

*The meaning of receiving an ESG (Environmental, Social, and Governance) integrated grade of “A” means that “we have adequately equipped the sustainable management system suggested by the governance, environment, and social standards, and there is little room for damage to shareholder value due to nonfinancial risks.” ESG rating of Korean firm provided by Korea corporate governance service (KCGS) (Kcgs, 2020), and the total ESG risk score data provided by Sustainalytics (Sustainalytics, 2020).

ISO37001: Certification of Anti-bribery management systems-Requirements with guidance for use, is a management system standard published by International Organization for Standardisation (ISO) in 2016.

G, only in headquarter website; L, low risk; M, medium risk; H, high risk; and CP, compliance program. A law-abiding system operated by the company to comply with laws and regulations related to fair trade. If the company disclosed a rating, the rating result filled it in parentheses.

#### 3.1.2 Domestic company

Of the seven domestic pharmaceutical companies that achieved KRW 1 trillion ($89.6 million) in domestic sales based on publicly available data in 2019, only one did not present a CSR section on its website. Those six pharmaceutical companies with CSR activities (?) introduced the compliance program (CP) and received an ISO 37001 (ANTI-BRIBERY MANAGEMENT SYSTEMS) certification, and one of them received an AAA grade in CP, which was reported as an excellent case because of its particularly high score in the social contribution area. An AAA grade in CP was related to the fact that Hanmi Pharm exhibits a CSR department. The mission, values, principles, and programs of social contribution activities were disclosed on their official websites; however, only four domestic pharmaceutical companies reported the results. Hanmi Pharmaceutical was the only company to write and disclose an ESG or a GRI report. Based on the ESG level evaluated by the Korea Corporate Governance Service, Hanmi Pharmaceutical exhibited the highest grade (A), and the rest were B or B+.

#### 3.1.3 Global companies

Among global pharmaceutical companies with a Korean branch, all companies with the top seven sales in 2019 presented a CSR section on the Korean branch website and introduced missions, values, principles, and CSR programs (Table 1). Two global pharmaceutical companies linked the code of ethics to the website of their headquarters, and the remaining five companies presented the CSR section on the website of their Korean branch. Researchers confirmed that the dedicated CSR team was located only at the global headquarters. Except for one global company, the results of CSR activities were disclosed on the website of the Korean subsidiary in the form of articles, press releases, number of persons, expenses, etc., and in the case of Novartis, the results were disclosed in detail only on the website of its global headquarters. All seven global pharmaceutical companies published GRI or ESG report on the website of their global headquarters. Unlike Korea, ESG ratings of global pharmaceutical companies are not standardized and have been published by various organizations (Escrig-Olmedo et al., 2010). For this analysis, the evaluation results of Sustainalytics cited by Yahoo Finance were used (Hale, 2016). Unlike the Korea corporate governance service (KCGS), which was evaluated in the order of A, B, and C, Sustainalytics calculated the ESG risk score and presented the individual total risk score and three grades of low, medium, and high, with the higher number indicating high risk of the ESG. Novartis demonstrated low ESG risk, Bayer demonstrated high ESG risk, and the remaining companies demonstrated medium ESG risk. However, this is the score of the global HQ, and regarding it as the score or activity of the Korean branch is difficult. In Table 1, the names of Korean pharmaceutical companies are coded from A to G.

### 3.2 Opinions of the stakeholders

From the perspective of global pharmaceutical companies, they argued that the development of innovative drugs, where no treatment is available, is a great social contribution as a philanthropic activity, and they also argued that consumers exhibit the same ideas based on the survey they funded (Lee et al., 2019). Some participants from global pharmaceuticals argued that R&D activities of pharmaceutical companies are not properly rewarded despite their large “social contribution” as a subsection of CSR. Also, they commented that programs, such as free medicines, also benefit patients greatly and should be evaluated as social contributions because companies bear a huge financial burden by conducting those activities. Next, they thought that donations related to the Catastrophic Health Expenditure (CHE) support could also be evaluated as philanthropic activities, which exceed economic, ethical, and legal responsibilities (Table 2).

**TABLE 2:** The key statement each participant.

Participants	“Can do”
Global pharmaceutical companies	• Development of innovative drug where no treatment
	• Free provision of medicines
	• Donations related to Catastrophic Health Expenditure (CHE)
Korean pharmaceutical companies	• Scholarship support for doctors are recognized as marketing activities
Consumer organization	• CHE support
	• R&D is an economic responsibility, not philanthropic activities
Patient group	• Free medicines (continuously)
	• Enable consumers to quickly take the drugs they need
	• Daycare centers and travel programs for severely ill patients

“The development of innovative medicines in the untreated areas is a great social contribution. In other words, I think that the development of drugs that are really in need is contributing to the domestic health industry” (global pharmaceutical companies).

“R&D activities are social contributions, and all benefits are delivered to patients. It just seems cruel only to the pharmaceutical industry” (global pharmaceutical companies).

On the other hand, participants from a domestic pharmaceutical company noted that programs, such as scholarship support for doctors, run by many pharmaceutical companies, are recognized as marketing activities and should not be evaluated as philanthropic activities (Table 2).

Consumer organizations, patient groups, and experts from academia argued that pharmaceutical companies’ R&D itself should not be considered as philanthropic activities. They said that it is an economic responsibility that a pharmaceutical company must do since the pharmaceutical companies collected all profits from the development of innovative new drugs and recent commercial success in pharmaceuticals actually related to cancer or orphan drugs (Table 2).

Also, they argued that granting a high drug price based on the value of philanthropy activities is inappropriate because the price consideration of the product unit and the philanthropic activity level of the company unit do not match. Consumer organizations and patient groups argued that R&D is considered a corporate economic and marketing activity.

The global pharmaceutical companies argued that donations related to CHE support are philanthropic contribution activities. However, the patient group thought that making donations was at the “ought to do” level of the CSR but not a philanthropic activity, which deserves a financial reward from the NHI.

Patients’ groups considered free medicines to be the most valuable philanthropic activity, but consumer organizations opposed the opinion of patient groups, and they were concerned that such activities would increase other consumers’ financial burden on drugs because the pharmaceutical industry might increase the price of other drugs to compensate for the donation. Next, the patient group replied that prompt access to drugs for those who need them is the philanthropic activities of pharmaceutical companies, and the operation of daycare centers and travel programs for severely ill patients are also a philanthropic activity.

“The donation of Catastrophic Health Expenditure (CHE) is considered to be included in philanthropic activities because it fits the reason for the existence of pharmaceutical companies” (Consumer organization).

“I think that support for Catastrophic Health Expenditure (CHE) should be excluded from the evaluation of philanthropic activity. It is inconvenient to be used as a means of receiving high drug prices through NIH fund by supporting disaster medical expenses” (Patient group).

“Free drug support is a philanthropic activity” (Patient group).

“The patient group may be positive with the free drug, but if so, the overall price of the other drug will increase, ultimately leading to negative consequences. This leads consumers to invisibly pay for expensive drugs” (Consumer organization).

### 3.3 Survey of consumer organization and patient society

The results of a written survey of consumer and patient organizations on the items whether they could be recognized as “can do” CSR activities of pharmaceutical companies are shown in Table 3.

**TABLE 3:** Whether pharmaceutical companies are recognized for “can do” level (philanthropic activities) by type of Corporate Social Responsibility activity.

Types of CSR activities	Consumer organization	Patient group
1) Donation to a severe disease disaster medical expense support project	X	X
2) Donation of medical expenses fund for people with rare and intractable diseases	X	X
3) Donated to the treatment fund for expensive anticancer drugs	X	X
4) Facility investment and know-how transfer when establishing a public pharmaceutical company	X	X
5) Free supply program for uncovered drugs	X	X*
6) Support for the patient’s out-of-pocket expenses for drugs with risk-sharing system	X	X
7) When the accessibility is improved by the first release of a new drug in the untreated field without alternative drugs in Korea	O	X
8) Activities to support the burden of caring for severely ill patients or caring for their families	O	X
9) Hospice support (expenses and services) for people with terminal disorders	O	X

The patient groups presented an opinion that none of the nine could be recognized as “can do” CSR activities yet rather as a “must” or “ought to” activities. The consumer organizations responded that when accessibility is improved by launching a new drug in untreated fields without alternative drugs for the first time in Korea, activities, such as support for the burden of care for critically ill patients and hospice support for terminally ill patients, can be recognized as CSR activities of pharmaceutical companies. However, on the basis of these activities, they were concerned about assigning a high drug price.

Through subjective responses, the consumer organization responded following philanthropic activities might deserve financial reward from NHI, thereby shortening the period of monopoly on new drug intellectual property rights or sharing intellectual property rights. The patient group responded that a program that supports the bereaved family members who died of the disease may fall into the abovementioned category.

## 4 Discussion

In this study, we reviewed the definitions and characteristics of the CSR activities of pharmaceutical companies and compared the CSR status of pharmaceutical companies between domestic and global companies. In addition, we solicited the opinions from stakeholders regarding the CSR activities of pharmaceutical companies and concluded that the definition of the philanthropic activities beyond the economic, ethical, and legal responsibilities varied greatly among stakeholders and failed to reach a social consensus in Korea. Because of the study, researchers confirmed that pharmaceutical companies operating in Korea, including global companies, prioritized the “must do” area of ethical management and the “ought to do” area of ESG management. Since Korean pharmaceutical companies lack in the areas of “must do” and “ought to do,” which are the minimum requirements of CSR, more efforts should be needed to equip the CSR activities that contribute to the development of genuine health care and the promotion of public health of consumers, including patients.

In Korea, the National Pension Service announced its participation in the Stewardship Code in 2018 and invested in 52 detailed indicators included in the ESG evaluation model, which evaluates nonfinancial factors, such as environment, society, and governance, that are considered for responsible investment (Kim and Koh, 2020). In March 2017, the Korea Exchange established an advanced disclosure service system related to corporate management transparency and governance structure through the detailed enforcement regulations of the disclosure regulations.

The pharmaceutical industry provides treatments for life-threatening diseases, but it exhibits the peculiarity of not being able to provide treatments to everyone at an affordable price (Ha, 2018). In Korea, treatments for rare diseases have already been able to receive benefits, such as a fast-track review system and conditional permit system of phase 3 (Kim et al., 2020). In addition, some companies with a large proportion of R&D have been certified as innovative pharmaceutical companies that can receive various benefits, such as tax assistance. This is a similar situation abroad, and 60 orphan drugs benefited through the US Orphan Drug Act. However, for these incentives, orphan drugs were criticized for sticking to high prices along with market monopoly rights.

In the policy forum on the social contribution activities of the pharmaceutical industry held at the National Assembly, academic experts said that the drug donation program is not a sustainable solution, as the company is not promising to donate drugs permanently or until new resources become available (Ha, 2018). They argued that the donation of medicine is important during emergencies or disasters, but its contribution is low in nonemergencies. The opinions of academic experts agree with the consumer organization and patient group statements from FGI. A government agency expert said that to establish a positive view on CSR, conducting it continuously and systematically rather than once is important. This opinion agrees with the findings from a case study on the CSR activities of pharmaceutical companies in Korea. Another government agency expert said that according to the Pharmaceutical Affairs Act, providing free medicines is illegal, but as a part of the CSR of pharmaceutical companies, he thought that free supply was a good way. However, it is judged that this opinion has not reached a social consensus. Also, the CSR experts pointed out that pharmaceutical companies should inform the reason why they do CSR to communicate social values, not marketing, and what value is realized through CSR. This opinion is in the same context as expecting CSR activities to voluntarily and actively donate profits, which are from patients, rather than receive high prices for new drugs in this FGI result. This study exhibits a limitation in that it did not perfectly recruit representative subjects. However, we think that the opinions of voluntary participants recommended through the stakeholder association are sufficiently saturated.

Witkowska, (2018) argued that the traditional CSR activities (philanthropy, community and neighborhood programs, volunteers, etc.) cannot be a rationale for high drug prices, which agrees with our results Witkowska, (2018). The authors argued that the true CSR of the innovative pharmaceutical industry is either abandoning patents or offering drugs for rare diseases at lower prices.

Our study showed that the CSR activities of Korean pharmaceutical companies at an early stage have just begun establishing a CSR system. Nevertheless, as one Korean pharmaceutical company exhibits a dedicated CSR team, receives the highest CP grade and ESG grade, and strengthens its sustainability, other companies try to benchmark them. Therefore, we can expect the CSR activities of pharmaceutical companies that meet global standards in Korea. In addition, although the headquarters of global pharmaceutical companies engaged in CSR activities at an international level, the content of social contribution to Korean society was unclear. Thus, deeper social consensus regarding financial incentives based on the CSR, especially philanthropic activities (“can do”) in Korea, is needed.

The social value of a pharmaceutical company’s CSR activities may vary from society to society, from culture to culture, and from era to era. Considering the situation of Korean pharmaceutical companies, where CSR activities are just taking root, the analysis results will be different 5–10 years from now. Next, we need continuous research to compare changes in the CSR activities of pharmaceutical companies and to draw social consensus by collecting stakeholder opinions.

### 4.1 Theoretical implications of this study

Through this study, we suggested to the Korean society that the CSR activities of pharmaceutical companies can be divided into three categories, and their value can be considered. This study is the first study to investigate and analyze the CSR activities of Korean pharmaceutical companies by applying the classification of CSR activities of pharmaceutical companies defined by (Leisinger, 2005) and (Carroll, 1999; Carroll and Buchholtz, 2014). In Korea, where the definition and value of the CSR activities in the pharmaceutical industry was unclear, this study guides further development of an evaluation framework related to the CSR activities of pharmaceutical companies. Next, the theoretical implication of our study is that social contribution values can be considered in the Korean society only when activities “must do” and “ought to do” are performed first and philanthropic activities are performed in the area “can do.” Furthermore, this study exhibits academic value in that it used a mixed methodology to examine whether theoretically suggested categories are acceptable in the real world.

### 4.2 Practical implications of this study

The Korean government received a request from pharmaceutical companies to reflect the value that the development of new medicines brings to the society in drug prices. The government began to wonder if it could be evaluated under CSR. To resolve this issue, we conducted research, including FGIs, with the stakeholder. According to (Bruyaka et al., 2013), the development of orphan drugs is no longer seen as a purely philanthropic activity, but it is also not a purely economic activity. However, the rapid increase in orphan drug development and marketing in countries, where orphan drug laws have been passed, indicates that the advantages from the laws act as incentives for development (Kotra, 2018). In Korea, regulations relating to orphan drug development are already stipulated in the Pharmaceutical Affairs Act, and incentives in that regard are found. Because of this study, the CSR activities of pharmaceutical companies in Korea were immature. Next, very little consensus existed among the stakeholders of the novel drug development as the CSR activity in the context of social contribution. Based on this study result, the request to assess the social contribution of novel drug development as a CSR activity and to take this into account in drug prices was not accepted by the government.

## 5 Conclusion

Because of this study, no consensus that the development of novel medicines should be recognized as a social contribution in the context of drug price review was found. Furthermore, we found that the CSR activities of domestic pharmaceutical companies are now in the early stages of establishing their system and those of multinational firms were mostly engaged in the headquarters, not Korean branches. Also, our analysis suggested that the pharmaceutical companies in Korea are trying to achieve the “ought to do” level (ethical responsibilities) while complying with the “must do” level (legal and economic responsibilities), yet not philanthropic activities. A social consensus regarding the philanthropic activities of pharmaceutical companies in Korea was not reached. While contemplating the sustainable management emphasized by the UN SDG, a strategy for the future wherein pharmaceutical companies, patients, and consumers will co-exist with each other should be established, based on the results of this study.

## Data Availability

The raw data supporting the conclusions of this article will be made available by the authors, without undue reservation.
